# Adapting and Pilot‐Testing a Training Program on Parental Cancer for Educational Professionals

**DOI:** 10.1002/cam4.71511

**Published:** 2026-01-11

**Authors:** Lene Johannsen, Wiebke Geertz, Laura Inhestern

**Affiliations:** ^1^ Department of Medical Psychology University Medical Center Hamburg‐Eppendorf (UKE) Hamburg Germany; ^2^ German Center for Child and Adolescent Health Hamburg Germany

**Keywords:** adolescents, children, educators, oncology, parental cancer, psychosocial support, school support

## Abstract

**Objective:**

Children and adolescents confronted with parental cancer are highly vulnerable. During such a family crisis, the social environment and external institutions (e.g., kindergarten, school) can play a crucial role in providing stability and support. This study aims to adapt and pilot‐test an existing training program on parental cancer for educational professionals.

**Method:**

This study is divided into three phases: (1) *conducting qualitative interviews* with parents with cancer, their children and educational professionals, (2) *adapting an existing training program* for healthcare professionals to the context of educational professionals, and (3) *pilot‐testing the training program* using quantitative and qualitative methods.

**Results:**

Interview results indicate that school is an important place to provide normality and stability. Parents appreciated it when educational professionals took the initiative by offering conversations, support and practical help. The adaptation resulted in a 3‐h training and was provided for *n* = 8 voluntary educational professionals. Preliminary results indicate that participants felt more knowledgeable, confident and empathic towards affected families after the training.

**Conclusion:**

Preliminary findings indicate that the training is a feasible approach to enhance knowledge and self‐confidence of educational professionals in dealing with families affected by parental cancer. To validate these findings from a small study sample, further research is needed.

## 1 Introduction

When a parent in a family is diagnosed with cancer, it inevitably affects the entire family system [1]. Every year, around 50,000 children and adolescents in Germany are affected by the situation of a parent being newly diagnosed with cancer [2]. Although the majority of children cope well with the situation, they are at risk for developing psychological problems [3, 4]. Affected parents are not only burdened by the disease and treatment itself, but also by insecurities and worries about their children [5]. Parents are often unsure if and how to tell their children about the diagnosis and worry about overlooking stress and changes in their children's behavior [5]. While many family routines are disrupted in the event of parental illness, the educational environment in kindergarten or school in particular can be an important source of support for children and adolescents [6, 7]. The maintenance of normality and routines can make a substantial contribution to the mental health of affected children [7]. Educational professionals (e.g., teachers, kindergarten nurses) can be important contacts for parents with cancer and can serve as an additional source of information about the child's condition and behavior. At the same time, uncertainties of educational professionals about how to deal and talk about cancer with affected parents and their children can lead to the topic of cancer being avoided and not addressed proactively [8].

Previous research indicates that, overall, the needs of children and adolescents are not always adequately addressed in the context of school, that some need more individualized information and support than is within the capacity or common knowledge of most teachers [9], and that there is a need for training in this area. While the role of educational institutions has been extensively investigated in the field of pediatric oncology [9, 10], little is known on this topic in the field of *children of parents with cancer*. Previous results indicate that school staff can benefit greatly from a training program and become more confident and less anxious about working with students and families facing parental cancer [8]. Hence, this study aims to:

- explore the needs and experiences with parental cancer in the educational context
- adapt an existing training program for oncology professionals on the topic of parental cancer to the context of educational professionals and
- pilot‐test the adapted training program regarding satisfaction and feasibility.

## 2 Methods

In the first phase, qualitative interviews were conducted with parents, children, and educational professionals. Based on these findings, we adapted an existing training program for oncology professionals to the educational context (phase 2) and pilot‐tested the training program including brief feedback on satisfaction and feasibility (phase 3).

### 2.1 Funding and Ethics

This study was funded by the Hamburg Cancer Society (Hamburger Krebsgesellschaft e.V.) and was carried out between March 2022 and April 2023. Ethics approval was obtained by the Local Psychological Ethics Committee of the University Medical Center Hamburg‐Eppendorf (LPEK‐0434). All participants received an information sheet with information about the study and provided written informed consent.

### 2.2 Data Collection and Procedure

#### 2.2.1 Qualitative Interviews

Parents and children/adolescents were recruited via existing contacts with local oncological institutions (e.g., psycho‐oncological outpatient clinic). Educational professionals were recruited via contacts of the research group. Interested study participants received an information sheet including study information. Individuals were only accepted as study participants once they had returned the signed consent form. For minor children, the consent of a parent was obtained.

L.J. (research associate) and I.B. (student assisstant) conducted interviews (in person or by phone) from May to November 2022. The guiding questions for the interviews were developed based on relevant literature and the research team's professional experience (Table 1) [11, 12]. The interviews were audio‐recorded for in‐depth analysis. Each person interviewed received an incentive in the form of a gift voucher worth €20.

**TABLE 1:** Central topics of the guiding questions.

Interviewees	Central topics
Parents	Experiences with the involvement of educational professionals in the context of (one's own) parental cancer Perceived hindering and facilitating factors regarding the involvement of educational professionals Support wishes according to educational professionals and institutions
Children/adolescents	Experience of dealing with parental cancer at school Wishes for educational staff in schools regarding support in the context of parental illness
Educational professionals	Experiences in everyday work with the topic of parental cancer Attitudes and knowledge according to parental cancer Perceived relevant skills for dealing with affected families Wishes and suggestions for a specific training program on parental cancer (e.g., how should the training program be designed in terms of format, duration and number of participants? What didactic aspects should it cover? What would you like the content to include?)

#### 2.2.2 Training Adaptation

The training program for educational professionals was adapted from an original training program on parental cancer that was developed in the context of a randomized controlled pilot study addressing healthcare professionals working in oncology [12, 13]. Two of the authors of this paper (L.I. and L.J.) were part of that research team. Detailed information on the development, the content and results of the pilot‐evaluation of the original training program has been published elsewhere [12, 13]. To adapt the original training program for an educational context (e.g., kindergarten and school), the results of the interviews with educational professionals, children and parents, as well as the expertise of the research team were used. The adapted training was designed to use a variety of didactic methods (e.g., lectures, discussions, audio‐visual materials and exchange of experiences).

#### 2.2.3 Implementation and Pilot‐Testing of the Training

The 3‐h training was delivered in February 2023 by two lecturers (L.I., L.J.) as a face‐to‐face format with nine voluntary educational professionals of one school in Hamburg, Germany. Two 10‐min breaks were taken during the training. To keep participants engaged throughout the training, lecturers asked participants at the beginning of the training about their work context, their experiences with affected families, and their motivation for attending the training. In the last module, the developed conversation guide was handed out to each participant.

The pilot‐testing of the training program included a post‐training questionnaire for all participants and qualitative follow‐up telephone surveys with part of the participants. The content of the questionnaire and example items are presented in Table 2. In addition, two open‐ended questions were included at the end of the questionnaire. The first asked what participants benefited most, and the second what they felt was missing. Follow‐up telephone surveys included questions about the extent to which participants' wishes and expectations regarding the training were met.

**TABLE 2:** The content of the post‐training questionnaires.

Section of the questionnaire	Example items
Personal information	Gender Age
Job‐related information	Current Occupation Amount of Work (e.g., full‐/part‐time)
Training feedback (5 point Likert Scale)	
Perceived changes in …	Knowledge about burden and needs of affected families Knowledge about possible reactions of children to a parental cancer disease Ability of understand the family's situation My own role as an educational professional in case of a parental cancer disease
Organizational aspects	Content and structure of the training Presentation format Pace at which the content was delivered
Specific training sections	Exchange between participants Conversation guideline
Atmosphere	Working atmosphere Preparation of lecturers Motivation of lecturers
Overall feedback	Recommendation of the training Training is suitable for the professional group

### 2.3 Sampling and Sample Size

For the interviews, we included *children* who were at least 11 years old and who had experienced parental cancer in the past. *Parents* were included if they had at least one minor child (< 18 years) at the time of the (partner's) cancer diagnosis, with no restrictions on cancer type or stage. Both ill parents and partners without cancer disease were included. *Educational professionals* were required to be employed at an educational institution. It was not necessary to have previous experience with parental cancer in the context of one's own educational institution.

The sample size for interviews was determined by the principle of theoretical saturation [14]. Data collection was conducted per subgroup until no new relevant themes or categories emerged from the interviews and there was sufficient material for meaningful analysis. The training program was designed as small group training. Accordingly, 6–8 educational professionals from one school were invited to participate in the pilot‐testing of the training. All training participants completed feedback questionnaires, and three participants took part in the follow‐up telephone survey.

### 2.4 Data Analysis

#### 2.4.1 Qualitative Data

Qualitative interviews and the follow‐up telephone surveys with educational professionals were analyzed by a primary coder (L.J.) based on Mayring's qualitative content analysis, using MAXQDA software [15]. A mixed inductive‐deductive approach was chosen for the analysis. The guiding questions in the interview guideline were used to create initial categories for the analysis (deductive approach). Additionally, inductive categories were formed from the transcribed interviews, and a first version of a coding scheme was developed by L.J. Afterwards, the coding was reviewed and discussed with a secondary coder (W.G., L.I.), resulting in a final coding schema that was applied to the full data set.

#### 2.4.2 Quantitative Data

Data on feasibility and satisfaction were analyzed with descriptive analyses using the statistical software SPSS version 27. Mean values and standard deviations were calculated for metric data (e.g., age, number of siblings) and frequencies were calculated for categorical and ordinal data (e.g., gender, diagnosis).

## 3 Results

### 3.1 Results From Qualitative Interviews With Parents, Children and Educational Professionals

The results of the interviews with parents, children and educational professionals on the role of educational professionals when a parent has cancer served as a basis for the adaptation of the training program to the educational context.

#### 3.1.1 Results From Qualitative Interviews With Parents

Qualitative analysis of the interviews with parents resulted in 4 categories: (1) *The perceived role of the educational institutions*, (2) *Professionals' behavior*, (3) *Parental needs*; and (4) *Communication about the illness with the educational professionals*.

##### 3.1.1.1 Sample Characteristics

We conducted 10 interviews with parents (7 mothers and 3 fathers) between 43 and 54 years (*M* = 47.7, SD = 3.5). Most of them were ill (*n* = 7, 70%) and had completed their school education with the general university entrance qualification (*n* = 7, 70%). Most of the interviewed parents were either married (*n* = 6, 60%) or living in a partnership (*n* = 2, 20%). For most of the interviewees, the parental disease occurred for the first time (*n* = 8) and all but one interviewee was no longer undergoing treatment at the time of the interview.

##### 3.1.1.2 The Perceived Role of the Educational Institutions

Parents reported that the educational institution served as a *constant* support during the illness and treatment and helped *to maintain normality*. Additionally, through educational professionals, there were people besides the parents who could help the children and provide emotional support. The kindergarten/school was seen as *a separate*, *unaffected space* for the children, which provided *distance* from the events and issues at home. At the same time, the childcare time also gave parents time for themselves.

I think it's really important that children have some kind of consistency. Because family life is completely turned upside down, and I think it's really important that they have a reliable, stable daily routine, but also emotional consistency. (E05)

##### 3.1.1.3 Professionals' Behavior

The behavior of educational professionals was rated as helpful when they were *proactive* by *offering conversations and support* on their own initiative, when they *provided practical help*, or when they *shared information* with their colleagues. Showing *understanding* for the family situation and *supporting the children* were also rated positively. In contrast, parents reported that a *lack of trust* or the feeling that the school and teachers were already *overburdened* had led to obstacles in communication. Some parents expressed their concern that their child and their child's behavior could be *reduced to the parental illness*.

I would have liked them to have said: “This is a normal child, and okay, the father died, that's one of the aspects of his biography”, but everyone has baggage to carry. (E010)

##### 3.1.1.4 Parental Needs

Parents expressed a need for *organizational support* (including flexibility regarding pick‐up and drop‐off times), the *proactive and repeated offer of help* (“Can we do something for you?”), and having *parent‐teacher meetings* (including feedback on the children's behavior at school or to discuss what can be communicated with whom about the parental illness).

[…] I think it is always nice for affected families and individuals when they receive offers. It shows that you have support […], you don't have to ask for it […]. (E05)

##### 3.1.1.5 Communication About the Illness With the Educational Professionals

Some of the parents stated that they had not explicitly discussed the illness with educational professionals. Reasons for this were, for example, parents and families were preoccupied with themselves, there was a lack of personal contact with the school, and parents were worried about a negative outside perception, were generally reserved, or because they did not perceive themselves as burdened. Other parents stated that they addressed the illness in an educational context because they wanted the educational professionals to have a closer look at the child, because they wanted to arouse understanding among educational professionals, or also to provide an explanation for the withdrawal from parental involvement, for example.

#### 3.1.2 Results From Qualitative Interviews With Children/Adolescents

Qualitative analysis of the interviews with children and adolescents, who have a parent with cancer, resulted in three categories: (1) *The role of the school and teachers' behaviour* (2) *Classmates' behaviour* and (3) *Information of the teacher about the parental disease*.

##### 3.1.2.1 Sample Characteristics

A total of seven children and adolescents were interviewed (*n* = 5 girls, *n* = 2 boys). The mean age was 13 years, with a range of 10–15 years. Most of the children had a sibling (*n* = 4).

##### 3.1.2.2 The Role of the School and Teachers' Behavior

Children and adolescents stated that school provided a place of *structure, routine, and normality* during the parent's illness, which was associated with *safety*.

So school was a structure that remained the same, which gave me a lot of security (K01).

The school was a *distraction* to get away from home and *to take one's mind off things*. Some children considered it helpful when teachers met them with *openness* and *proactivity*, offering support and conversations, as the children and adolescents sometimes did not dare to do so themselves.

Then I think it's better if the teacher approaches the pupil, because I think that many don't dare or think that it's strange if I approach them myself. (K05)

*Acceptance* and *understanding of individual needs* are also reported as helpful ways of dealing with them. The children's statements indicated that their autonomy must be respected and that it is important for them to decide what help to seek or who to tell about the illness.

[…] but that the teachers don't force children, so that the children still decide to do it themselves, because I don't think there's any point in forcing someone, so to speak. Most children are old enough to say “I don't want to do this” and then they don't do it. (K01)

Even though only a few children reported *having agreements with their teachers* regarding practical help in everyday school life, this is seen as helpful. For example, children wanted to be allowed to *leave class on their own responsibility* if they were not feeling well. At the same time, children stated that they liked it when teachers *behaved in a normal way* (despite their parents' cancer diagnosis), when they (the children) were *not favored* and therefore *did not take on a special role*.

… because I don't think that [parental cancer] is a privilege or a reason to be treated differently. (K05)

##### 3.1.2.3 Classmates' Behavior

Regarding classmates, children considered it positive when they *took the initiative and included, supported, distracted, or cheered them up*. Some reported that they talked to other children who had also experienced a close relative suffering from cancer. It also emerged that some children felt they had taken on a special role and were not being understood in their particular situation. They were *worried about rejection* and negative judgment from others and thus also worried about expressing their emotions to others.

And I always had the feeling when I talked about it that I, for example, people thought I wanted attention or something, which I never intended to achieve with it. (K05)

##### 3.1.2.4 Information of the Teacher About the Parental Disease

Teachers were mostly informed by a parent or the child themselves. The reasons given by parents for informing teachers were that they wanted the teachers to know in general and that they wanted teachers to have an explanation for possible unusual behavior at school or incomplete tasks. Some children and adolescents also stated that this made them feel more seen and recognized in their situation and that they found it reassuring and relieving that they did not have to explain themselves. One child reported that he did not care whether the teachers knew about the illness. Several barriers to communication with teachers were reported: some children perceived the teacher as a stranger, wanted the topic of the illness to remain within the family or stated that they were not shown enough sensitivity by the teachers. When informing the teachers, some interviewees also reported that it was important for them to decide for themselves whether and to whom they wanted to talk about the parents' illness.

#### 3.1.3 Results From Qualitative Interviews With Educational Professionals

Qualitative analysis of the interviews with educational professionals resulted in two categories: (1) *The role of educational institutions and professionals*; (2) *Working with affected families*.

##### 3.1.3.1 Sample Characteristics

Eight educational professionals from educational institutions (7 women, 1 man) participated in the interviews. Professionals were between 20 and 59 years old (*M* = 42.5, SD = 11.2). Half of the interviewees stated that they were employed at a secondary school (*n* = 4); three people reported working at a primary school, and one person worked at a special needs school. All participants stated that they had already experienced cancer in their personal environment.

##### 3.1.3.2 The Role of Educational Institutions and Professionals

Interviewees stated that they would consider the educational institution to be an *important, reliable structure* for the children in the event of a parental cancer, *providing everyday life, normality, and safety*.

I think it's important that they know the dependability of the institution, that they know “it will always go on here, I have the structure here, I can hold on to it”. (P04)

Educational professionals stated that they often saw themselves in the role of *mediators for support services* and that they would *differentiate their own professionalism* and area of responsibility from that of psychological/psychotherapeutic professionals. *Maintaining one's own boundaries* is also an important factor for many professionals.

You have to recognize your own limitations and then make sure you say, “Now, I'm going” to bring in the professionals'. I think that's always important. (P02)

##### 3.1.3.3 Working With Affected Families

In their behavior towards parents, it was particularly important for individual professionals to *behave according to the wishes of the parents or family* and to *accept* and *respect* their needs. At the same time, educational professionals would consider working with the parents to be relevant; for example, by *offering parent‐teacher conversations*. The aspect that it is important to make arrangements with parents in order to *clarify what may be communicated* and shared about the family situation was also emphasized. The perceived importance of respecting and accepting the individual needs of the family was also evident in interactions of educational professionals with the children. Interviewees emphasized that they did not want to impose themselves and that their *own behavior had to be adapted according to the personality and needs of the respective child*. They further reported they could only offer themselves as a *contact person* and a *person of trust* in the sense of a companion in order to be able to provide support. They stated that they consider it important to *show understanding and empathy* for the children and the family situation, *to offer conversations, to take responsibility* for the child, but at the same time *not to put the child in a special role* and *not to limit them to the parental cancer*.

[…] and see if the child even wants [to talk] and if it is ready for it, s I would never impose myself. (P03)

### 3.2 Results of the Training Adaptation for the Context of Educational Professionals

**FIGURE 1:**
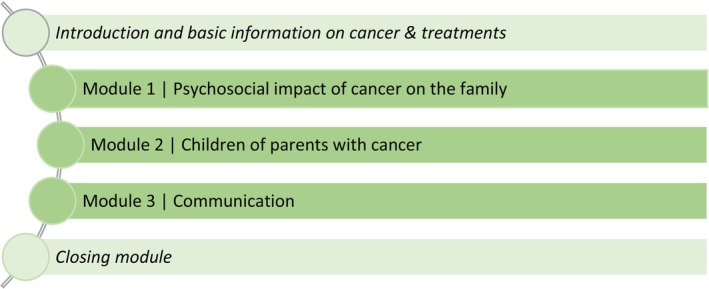
Overview of training content.

The training adaptation resulted in a 3‐h training comprising three modules (Figure 1), focusing on the disease's psychosocial impact on the family, children's wishes for support and possible age‐related reactions to parental cancer disease, and communication within the family and between educational professionals and families. As educational professionals do not have basic knowledge of cancer diseases and treatments (unlike oncology professionals), fundamental information on cancer (e.g., prevalence rates, most common tumor types by gender) and treatment (e.g., chemotherapy, radiation therapy) was taught at the beginning. Furthermore, as a result of the interviews with educational professionals, we added a section on “Conversation With Parents About Parental Disease” as educational professionals told to be unsure how to communicate with parents about their (partner's) disease and wished for guidance and support.

**FIGURE 2:**
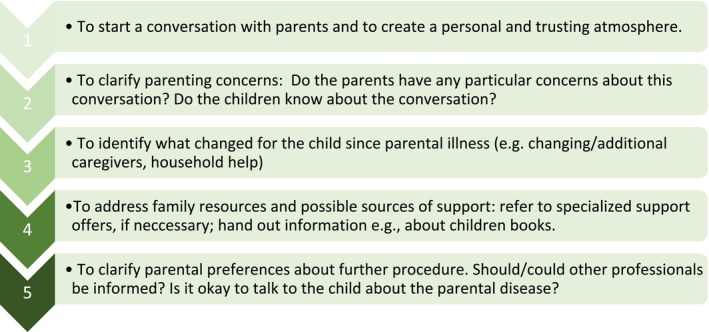
The rough structure of the conversation guide for educational professionals in talking with parents affected by cancer.

In addition, based on the results of interviews with educational professionals, we developed a *conversation guide* to give educational professionals an orientation on how and what to talk to parents affected by cancer (Figure 2). The rough structure is based on the basics of communication skills (e.g., how to start a conversation in a way that creates a personal atmosphere) and is supplemented with specific topics that may be relevant to parents.

Furthermore, we included a section on “How to Deal With Children and Adolescents Having a Parent With Cancer?” Therefore, we used literature, the research team's expertise, and the results of the interviews with children and adolescents to formulate basic advice on how to deal with children affected by parental cancer (e.g., offering children the opportunity to talk without forcing; providing practical support).

### 3.3 Results of the Pilot‐Testing

After the training, all participants received the feedback questionnaire (*n* = 9). All but one of the participating professionals were female (*n* = 8, 89%) and the participants were on average 44 years old (range 33–57 years, SD = 7.4). Three teachers, two educators (German ‘Erzieher:in’), one social and one special educator (‘German Sozialpädagog; in/Sonderpädagog:in’) and two people trained as both teachers and special educators (German ‘Sonderpädagog:in’) participated.

#### 3.3.1 Quantitative Feedback

Quantitative feedback results are based on data from *n* = 9 participants. Both the organization and the structure of the training were rated positively (Tables 3 and 4). In a free‐text box, all respondents gave information on what they benefited most from in the training: the information on possible reactions of children to parental disease (*n* = 4), the conversation guide (*n* = 3), the given basic knowledge about cancer (*n* = 1) and the lecturers' examples from clinical practice (*n* = 1). Three respondents indicated what they missed most in the training: additional exchange on own case examples, information on how to handle the situation with class‐mates/peer‐group and how to deal with parents who do not want to talk.

**TABLE 3:** General feedback on the adapted training.

	*M* (SD)
Organization and structure	
The content and structure of the training were comprehensible and logical	3.9 (0.3)
The form of the presentation was appropriate	3.7 (0.5)
The scope and depth of the topics covered were appropriate	3.6 (0.5)
The pace of the content taught was appropriate	4 (0.0)
The training was structured and organized	4 (0.0)
There was enough room for questions and comments from the participants	3.8 (0.4)
The level of involvement of participants was pleasant and appropriate	3.9 (0.3)
The length of the training was appropriate	3.8 (0.4)
Evaluation of individual training elements	
The contents were presented in an understandable way	3.9 (0.3)
The exchange was inspiring	3.3 (0.5)
I find the conversation guide helpful for discussions with affected parents	3.9 (0.3)
I can well imagine using the conversation guide for future conversations with parents	4 (0.0)
Overall rating	
I would recommend the training to others	3.9 (0.4)
Overall, I am satisfied with the training	3.9 (0.3)
I found the training suitable for my professional group	4 (0.0)

*Note:* Rating on a 4‐point Likert‐Scale (1 = in no case, 4 = in any case; additional response option: ‘I don't know’). *M*, mean; SD, Standard Deviation; *n* = 9.

**TABLE 4:** Feedback on perceived changes after the training.

The training enables me to …	M (SD)
… increase my knowledge on burden and needs of affected families	4.0 (0.0)
… increase my knowledge on possible reactions of children to parental disease	4.0 (0.0)
… better empathize with the situation of affected families	4.0 (0.0)
… feel prepared to talk to parents about their illness and the effects in the pedagogical context of the child	3.6 (0.53)
… become clearer about ways of supporting affected families	3.7 (0.5)
… become more aware of my own role as an educator in the event of parental cancer	3.4 (0.53)
… feel more confident in talking to affected parents	3.6 (0.52)
… feel more empathetic towards affected families	3.6 (0.53)
… feel more confident in identifying stress reactions in children	3.5 (0.53)
I consider the content of the training to be relevant to my everyday school life	3.6 (0.53)

*Note:* Rating on a 4‐point Likert‐Scale (1 = in no case, 4 = in any case; additional response option: ‘I don't know’). *M*, mean; SD, standard deviation. Study sample varies between *n* = 8 and *n* = 9, since some items were not answered by all respondents.

#### 3.3.2 Qualitative In‐Depth Feedback

Approximately 2–3 weeks after the training, short telephone surveys were conducted with *n* = 3 training participants to assess the feedback in more detail. The telephone surveys took between 11 and 19 min. All respondents were female. All participants reported that they were very satisfied with the training. One interviewee told that he/she was motivated to participate in the training since *the topic of parental illness seems to be part of teachers' professional work life but is not part of the teacher training*. Participants reported that they felt *well involved* in the training to a good extent, that the *balance between lecturer input and group discussions was good* and that they had benefited from the materials brought along by the lecturers (including literature for families with parental cancer). Some participants expressed a *wish for the training to be repeated* and offered to educational professionals on a *regular basis*.

All participants reported that they would like a second, *more in‐depth block* in addition to the three‐hour training session. This could either be used to refresh what they had learnt following a certain period of time or to make even more specific reference to how to deal with the topic in a pedagogical setting. Participants also wished for an extra section as a kind of ‘best practice’ and/or to work with their own case examples in role plays.

## 4 Discussion and Conclusion

### 4.1 Discussion

The findings of the qualitative interviews with parents, children and professionals in the first study phase indicate the relevant role of educational professionals when a parent has cancer. Based on participants' feedback, the adapted training can be a promising approach to enhance professionals' knowledge, to reduce uncertainties and to facilitate open and proactive communication with affected families. However, the small sample size of this study only allows for preliminary results. Further research is needed to validate the findings.

The qualitative findings indicate that parents and children value the school as a place to distract and to maintain normality, educators as possible additional emotional or practical support. They perceived proactive school staff as helpful and wished for acceptance and understanding of their individual needs. Parents also reported a lack of trust, seemingly overwhelmed teachers, or fear that their child's behavior could be attributed to the parent's illness, which led to a reluctance to communicate. Children and adolescents reported fears of rejection and negative judgment by others and the resulting reluctance to talk openly about it. Educators saw their role as a possible contact person that behaves according to the wishes of parents and children, accepting and respecting their needs, with the importance to clarify what may be communicated with the school and with the child.

Although it is difficult to compare school systems from different countries, teachers' statements of our study are similar to those of school nurses recorded in a qualitative study from Sweden [16]. School nurses reported a lack of structured routines about how to find out that a parent of a child at their school is seriously ill and the condition for being able to care for the child is that the support is provided in agreement with the parents. Therefore, we addressed this in the developed conversation guide of the training: educational professionals should clarify the preferences of parents regarding dealing with the family's situation in school. Proactive behavior on the part of professionals can help to clarify whether and how they can support the affected family.

Taking into account the pilot nature of the study, the present results indicate that the training course may be an appropriate means to strengthen the knowledge of educational professionals on the topic and to reduce uncertainties in dealing with affected families to promote proactive and open communication. This is in line with the findings of a study by Fasciano et al., who developed and piloted a program to educate school professionals in the USA about the impact of parental cancer on families [8]. The program's content was comparable to that of our study. In the study of Fasciano and colleagues, participants completed self‐report questionnaires before and after the training. Pre‐post analyses showed that participants rated themselves as more knowledgeable and confident in their ability to support families affected by parental cancer following the training [8]. A particular aspect within the school context is the identification of children and adolescents who are engaged in caring for their ill parent (young carers) [17, 18]. Research findings indicate that young carers may experience problems in concentrating in class, failure to complete homework, absenteeism and school dropout [19, 20]. Therefore, it is crucial that educational professionals are aware of this phenomenon and are sensitized to identify children and adolescents that are young carers [21, 22].

#### 4.1.1 Study Limitations

As study participation was voluntary, a selection bias in terms of the participation of particularly interested and motivated professionals cannot be ruled out. The training was conducted with people who belonged to the same educational institution and it is unclear to what extent the results can be transferred to professionals from other institutions. In addition, the training was pilot‐tested with a small sample of educational professionals and the post‐training questionnaires were based on self‐report only. We are not able to make any statements about real changes in the behavior of the professionals and can only provide descriptive statistics. Post‐training in‐depth interviews were conducted with only three volunteers. Further research is needed to validate the findings.

#### 4.1.2 Clinical Implications

Educational institutions can play a crucial role in providing stability and support for children in case of parental cancer. Parents can be relieved by the fact that educational professionals keep an eye on their children at school. Nevertheless, educators should be well equipped to meet the needs of parents and children in a sensitive way and to feel confident about dealing with this situation. By empowering educators, the support system of affected families can be strengthened.

### 4.2 Conclusion

This interview and pilot study highlight the relevance of the educational environment in case of parental cancer. Educational professionals can support children and families and school is a key for maintaining routines. Comprehensive basic training for educational professionals on the topic of “parental cancer” could make a significant contribution to provide adequate support for affected parents and their children/adolescents by trained professionals acting as contact persons in educational institutions.

## Author Contributions

**Lene Johannsen:** conceptualization (equal), data curation (equal), formal analysis (equal), investigation (equal), methodology (equal), project administration (equal), writing – original draft (equal). **Wiebke Geertz:** conceptualization (equal), data curation (equal), methodology (equal), writing – review and editing (equal). **Laura Inhestern:** conceptualization (equal), investigation (equal), methodology (equal), project administration (equal), resources (equal), supervision (equal), writing – original draft (equal), writing – review and editing (equal).

## Funding

This study was funded by the Hamburger Krebsgesellschaft e.V. (Hamburg Cancer Society). Laura Inhestern holds a professorship endowed by the Kindness for Kids foundation.

## Conflicts of Interest

The authors declare no conflicts of interest.

## Data Availability

The participants of this study did not give written consent for their data to be shared publicly, so due to the sensitive nature of the research, supporting data is not available.
